# Transcatheter Aortic Valve Replacement: Current Status and Future Indications

**DOI:** 10.3390/jcm13020373

**Published:** 2024-01-10

**Authors:** Manish Vinayak, Pier Pasquale Leone, Richard Tanner, Vishal Dhulipala, Anton Camaj, Rakhee Rajendra Kumar Makhija, Amit Hooda, Annapoorna S. Kini, Samin K. Sharma, Sahil Khera

**Affiliations:** Mount Sinai Heart, Mount Sinai Hospital, New York, NY 10029, USA; pierpasquale.leone@mountsinai.org (P.P.L.); richard.tanner@mountsinai.org (R.T.); vishaldhulipala@gmail.com (V.D.); anton.camaj@mountsinai.org (A.C.); rakheerajendrakuma.makhija@mountsinai.org (R.R.K.M.); amit.hooda@mountsinai.org (A.H.); annapoorna.kini@mountsinai.org (A.S.K.); samin.sharma@mountsinai.org (S.K.S.)

**Keywords:** aortic stenosis, aortic valve replacement, transcatheter aortic valve replacement

## Abstract

In the past two decades, transcatheter aortic valve replacement (TAVR) has transformed the management of aortic stenosis and has become the standard of care regardless of surgical risk levels. Advances in transcatheter valve design across newer generations, improved imaging, greater operator expertise, and technical enhancements have collectively contributed to increased safety and a decline in procedural complications over this timeframe. The application of TAVR has progressively expanded to include younger patients with lower risks, who have longer life expectancies. This article offers an up-to-date review of the latest innovations in transcatheter delivery systems, devices, and its possible future indications.

## 1. Introduction

Since the first-in-man procedure in 2002 [1], transcatheter aortic valve replacement (TAVR) has revolutionized the management of symptomatic severe aortic valve stenosis (AS). The rapid technological advancement of transcatheter prostheses, patient selection and increased operator experience have led to a paradigm shift in TAVR performance, expanding this less-invasive treatment to a larger patient population [2]. As TAVR is increasingly offered to younger, lower-risk patients with an increased life expectancy, issues such as transcatheter heart valve (THV) durability, coronary access, and redo-TAVR are clinically pertinent issues needing consideration [3]. In this article, we aim to review the currently available transcatheter delivery systems and devices, limitations, and challenges associated with TAVR, and the possible future indications.

## 2. Current TAVR Indications

**Figure 1:**
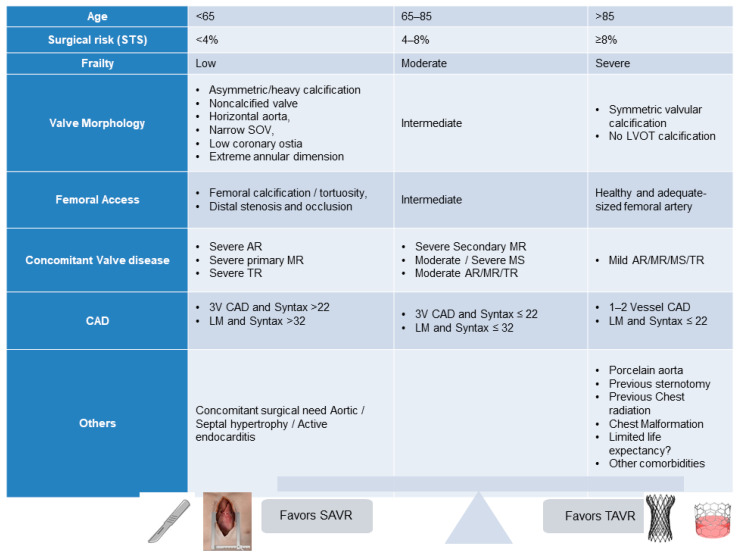
Patient’s selection: Various clinical and anatomical factors affecting the choice between TAVR and SAVR [6].

The safety and efficacy of TAVR has been well established in patients with severe AS in a series of randomized clinical trials across the entire spectrum of surgical risk. Both the American College of Cardiology/American Heart Association (ACC/AHA) and European Society of Cardiology (ESC) guidelines for the management of patients with valvular heart disease have been updated and provide recommendations; however, the clinical decision between TAVR vs. SAVR requires the consideration of multiple and complex clinical and anatomical factors (Table 1, Figure 1) [4,5].

## 3. Current TAVR Devices

**Figure 2:**
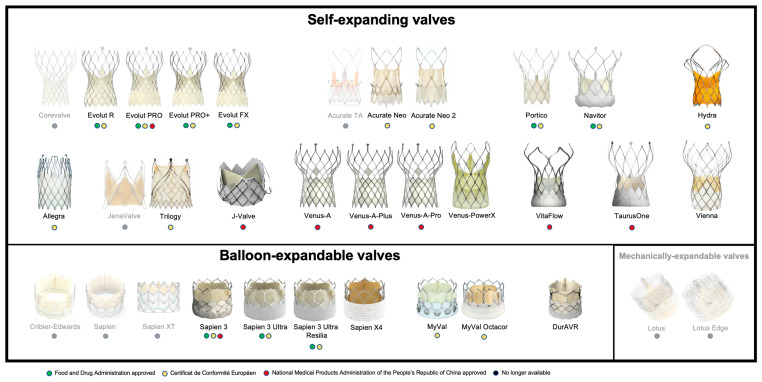
Currently available TAVR prosthesis.

As TAVR is being increasingly adopted across the globe [2], and new THV, as well as new THV iterations, are being developed. The main design classification applied to THV is in regard to the mechanism of valve expansion, and includes self-expanding valves (SEV), balloon-expandable valves (BEV) or mechanically expandable valves (MEV), as shown in Figure 2 and Table 2.

### 3.1. Self-Expanding Valves

The Evolut FX (Medtronic, Minneapolis, MN, USA) is the latest iteration from the CoreValve family of valves [7,8]. The long nitinol frame and its relatively small diamond-shaped cells (approximately 12 French) impact the feasibility of selective coronary access. The delivery catheter, EnVeo InLineTM sheath, replaces the need for a separate introducer sheath. The replacement of the double spine with a single-spine shaft has improved the delivery system flexibility and valve deliverability [9].

The Acurate Neo 2 (Boston Scientific, Marlborough, MA, USA) received Conformité Européen (CE) mark in 2020, while the Acurate IDE trial (NCT03735667) has an estimated primary completion date in early 2024. Axial stabilization arches above the leaflet level, with a large open space in between them and an upper crown, allow for easier coronary access and reduce the risk of coronary obstruction. Acurate Prime XL is an iteration of ACURATE neo-2, and is designed to treat a larger native aortic annulus diameter, ranging between 26.5 mm and 29 mm. The valve is compatible with a 14F sheath transfemoral delivery system that features a flexible catheter and distal release mechanism to allow for quicker valve release in a single motion [10].

The Portico (Abbott, Chicago, IL, USA) is the first intra-annular SEV to have obtained Food and Drug Administration (FDA) approval in the United States. Its large frame cell design (13.5–20.8 Fr according to valve size) simplifies coronary access. The annular porcine pericardial cuff was replaced by the NaviSealTM active sealing cuff in its new iteration, the Navitor valve (Abbott, Chicago, IL, USA), which received CE mark in 2021 and FDA approval in 2023. The Navitor Titan valve (35 mm) enlarged the range of treatable annuli with this valve (up to 30 mm in diameter) [11].

Hydra (Sahajanand Medical Technologies Limited, Mumbai, India) has three tentacle-like components to optimize alignment and conformability to the aorta, and large open frame cells (>15 French) facilitate easier coronary access. Domestic THVs in China aimed at treating bicuspid aortic valves share the common feature of high radial force and include the Venus-A (Venus Medtech Inc., Hangzhou, China), VitaFlow (MicroPort, Shanghai, China) and TaurusOne (Peijia Medical, Suzhou, China). Allegra (New Valve Technology, Hechingen, Germany) includes a Permaflow feature, which maintains flow during implantation, with the intent to abolish the need for rapid pacing. Lastly, dedicated prostheses were designed to treat patients with isolated aortic regurgitation by the engagement of native aortic valve leaflets, including the Trilogy (JenaValve Technology GmbH, Munich, Germany) and J-Valve (JC Medical Inc., Burlingame, CA, USA). Details of SEV are reported in Table 2.

### 3.2. Balloon-Expandable Valves

The Sapien 3, Sapien 3 Ultra, and Sapien 3 Ultra Resilia (Edwards Lifesciences, Irvine, CA, USA) are the third, fourth, and fifth iterations within the Sapien family, respectively, and are currently the only FDA-approved BEV. A textured polyethylene terephthalate outer cuff, increased in height by 40% in the Sapien 3 Ultra, low stent frame height, and large open-cell configuration of the upper frame allow for PVL risk mitigation and often straightforward coronary artery access. RESILIA leaflet tissue [12], along with a novel frame design that enables adjustable sizing in 0.5 mm increments, and independent valve rotation control enabling commissural alignment are new features included in the latest iteration, Sapien X4, not commercially available yet as of December 2023. The ongoing ALLIANCE study will assess the safety and efficacy of Sapien X4 in all surgical risk categories including the bicuspid and valve-in-valve patients [13].

The unique feature of MyVal and MyVal Octacor (Meril, Vapi, Gujarat, India) is that they are available in nine different sizes, each 1.5 mm apart, ranging from 20 mm to 32 mm, allowing for the treatment of patients across a wide range of annular dimensions [14].

DurAVR (Anteris Technologies, Toowong, Australia) is the first biomimetic THV undergoing an early feasibility study, and promising 30-day forward-flow hemodynamics data have been recently revealed [15].

### 3.3. Mechanically Expandable Valves

Lotus and Lotus Edge (Boston Scientific, Marlborough, MA, USA) THVs, characterized by a braided nitinol frame, very high radial strength, and adaptive seal, were the only MEVs available until their recall in late 2020 due to delivery system issues.

## 4. Valve Durability

Similar to the surgical bioprosthetic valves, transcatheter heart valves (THVs) have potential to fail and this issue is particularly pertinent in younger, less co-morbid patients who are likely to outlive the valve’s longevity.

The studies evaluating the mid-term durability of THVs from the PARTNER-1, CoreValve US Pivotal, SURTAVI-IR, NOTION trials have shown stable mean aortic valve gradients and a low incidence of valve degeneration at 5 years’ follow-up [16,17,18,19]. Among the low-risk patient population, at 2 years and 5 years, the follow-up data of PARTNER-3 showed similar mean gradients and effective orifice areas after TAVR versus surgery (12.8 ± 6.5 vs. 11.7 ± 5.6 mm Hg; 1.9 ± 0.5 cm^2^ vs. 1.8 ± 0.5 cm^2^) [20,21]. Similarly, the 4-year follow-up data of the EVOLUT-LR trial showed that the patients in the TAVR group had sustained improvement in hemodynamics as measured by echocardiography, with significantly lower aortic valve mean gradients and greater effective orifice area with no difference between the groups in moderate or greater PVR [22]. Recently, Jorgensen et al. reported the 10-year follow-up data from the NOTION trial, which included relatively younger patients with lower surgical risk (mean age 79.1 ± 4.8 years and a mean STS predicted risk of mortality score of 3 ± 1.7%), which showed a significantly lower rate of SVD (20.2% vs. 37.3%, *p* < 0.05), a greater effective orifice area, and a lower transvalvular gradient in the TAVR group compared to SAVR [23]. Furthermore, ex vivo bench studies have demonstrated favorable hemodynamics simulating up to 25 years of use for the balloon-expandable Sapien 3 THV and the self-expanding ACURATE neo 2 THV (Boston Scientific Corporation, Natick, MA, USA) [24,25].

To address the issue of THV durability, intraprocedural strategies to optimize valve expansion such as predilatation, appropriate valve sizing, avoidance of postdilatation and newer bioprosthetic platforms including modifications in the leaflet material and designs have been developed which may improve leaflet longevity and THV durability [24,25].

### 4.1. RESILIA Tissue

Structural valve degeneration (SVD) can be caused by a buildup of calcium that may impact the long-term durability of bioprosthetic valves. RESILIA tissue (Edwards Lifesciences) involves the stable capping of free aldehydes offering enhanced anti-calcification technology, preventing calcium binding and glycerolization while simultaneously allowing dry storage that simplifies leaflet handling while preserving and protecting the leaflet tissue. RESILIA tissue has been priorly used in Edwards surgical valves (INSPIRIS RESILIA aortic valve, MITRIS RESILIA mitral valve, and KONECT RESILIA aortic valve conduit, Edwards Lifesciences). The recently presented 7-year follow-up data of the COMMENCE trial demonstrated low rates of SVD (99.3% freedom from SVD) with a stable hemodynamic performance and minimal regurgitation [26]. Currently, RESILIA tissue is being used in the SAPIEN 3 Ultra RESILIA valve and will be incorporated in the next generation of the SAPIEN X4 THV platform which may further enhance the valve durability and longevity [12].

### 4.2. DurAVR THV System (Anteris Technologies)

DurAVR™ THV is a novel first-in-class biomimetic balloon-expandable valve made to particularly address the challenges related to the THV durability and hemodynamic performance. DurAVR valve is made from a single piece of tissue which has been designed and molded to mimic the anatomy and performance of a native human aortic valve. This is distinct to all the other commercially available THVs that consist of three separate leaflet tissues sewn onto a valve frame. Detailed computational fluid modeling comparing the two leaflet designs has revealed that the single-piece leaflet design facilitates optimal laminar flow which results in the uniform distribution of the leaflet stress and hence minimizes leaflet strain and prolongs durability. This results in a more uniform distribution of leaflet stress, facilitating optimal laminar flow and minimizing leaflet strain. Furthermore, this unique leaflet design offers a larger coaptation area and improved coaptation length, reducing the risk of leaflet pin-rolling. Notably, the DurAVR THV also utilizes the novel ADAPT tissue-engineered anti-calcification technology, employing DNA- and glutaraldehyde-free acellular bovine pericardial leaflet tissue. This decreases the risk of leaflet calcification and structural valve deterioration (SVD), contributing to the enhanced durability and longevity of the DurAVR THV System. Additionally, the unique leaflet design allows for a shorter stent height with a spacious open cell geometry, and the ComASUR™ delivery system provides controlled deployment, potentially increasing the likelihood of achieving commissural alignment and facilitating coronary access [15].

In the first-in-human, prospective, single-arm, single-center study designed to assess the safety and feasibility of the DurAVR THV in 20 patients, procedural success was 100%, with no device-related complications. The DurAVR demonstrated superior hemodynamics, with an effective orifice area (EOA) of 2.36 cm and a mean gradient of 7.8 mm Hg. These hemodynamic effects were sustained at the 1-year follow-up in the first 12 patients. Furthermore, the CMR testing in the five patients matched to healthy controls showed that DurAVR maintains laminar aortic flow [15].

## 5. TAVR in Bicuspid Valve Anatomy

Bicuspid aortic valve (BAV) is the most common congenital valvular heart disease affecting 1 to 2% of the United States’ population, and accounts for about 50% of the patients requiring aortic valve intervention [27]. BAV patients were excluded from the pivotal randomized controlled trials (RCTs) due to potential anatomical challenges such as asymmetric and higher leaflet calcification, fused raphe, larger annulus size, and associated aortopathy. Early TAVR experiences with first-generation transcatheter heart valves (THVs) in bicuspid aortic stenosis (AS) patients reported worse in-hospital outcomes, an increased incidence of PVL, device malpositioning, permanent pacemaker implantation, aortic root injury, and stroke. The improvement in device technology, imaging techniques, better understanding of BAV anatomy, and growing operator experience has led to greater procedural success and better clinical outcomes. From data in the low-risk subgroups, in the PARTNER 3 Bicuspid Low-Risk registry, SAPIEN 3 THV in BAV anatomy performed similarly to a matched cohort of patients with tricuspid AS patients, in terms of the 1-year primary endpoint of death, stroke, and cardiovascular rehospitalization [28]. Yoon et al. reported calcified raphe and excess leaflet calcification as independent predictors of 2-year mortality after TAVR in BAV anatomy, with an incremental risk if both were present [29]. Hence, the identification of high-risk BAV phenotypes guides the heart team in decision-making for evaluating the suitability of TAVR vs. SAVR.

## 6. Valve-in-Valve

**Figure 3:**
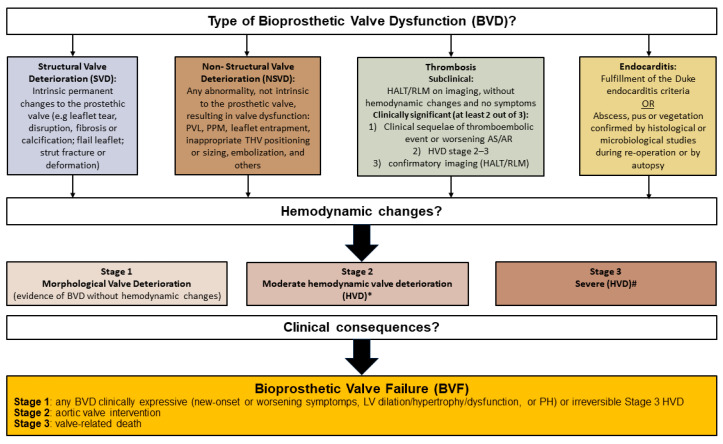
Classification of bioprosthetic valve dysfunction (BVD) and bioprosthetic valve failure (BVF) [30]. * Moderate HVD: Increase in mean trans-valvular gradient ≥ 10 mmHg resulting in mean gradient ≥20 mmHg with concomitant decrease in EOA ≥0.3 cm^2^ or ≥25% and/or decrease in Doppler velocity index ≥0.1 or ≥20% compared with echocardiographic assessment performed 1–3 months post-procedure, OR new occurrence or increase of ≥1 grade of intra-prosthetic AR resulting in ≥ moderate AR. **# Severe HVD**: Increase in mean transvalvular gradient ≥20 mmHg resulting in mean gradient ≥30 mmHg with concomitant decrease in EOA ≥0.6 cm^2^ or ≥50% and/or decrease in Doppler velocity index ≥0.2 or ≥40% compare with echocardiographic assessment performed 1–3 months post-procedure, OR new occurrence, or increase of ≥2 grades of intra prosthetic AR resulting in severe AR. AR: aortic regurgitation; AS: aortic stenosis; HALT: hypo-attenuated leaflet thickening; LV: left ventricle; PH: pulmonary hypertension; RLM: reduced leaflet motion.

Valve-in-valve (ViV) TAVR has emerged as a less invasive alternative for the treatment of failed bioprosthetic aortic valves. To standardize the definition of valve durability, a consensus statement has been proposed by the European Association of Percutaneous Cardiovascular Interventions (EAPCI), the ESC and the European Association for Cardio-Thoracic Surgery (EACTS), and by the Valve Academic Research Consortium-3 (VARC) [30]. The main etiologies for bioprosthetic valve dysfunction (BVD) of transcatheter and surgical bioprosthetic aortic valves are depicted in Figure 3.

### 6.1. Surgical Bioprosthetic Aortic Valves

Surgical bioprosthetic aortic valves can be classified according to the type of frame (stented, stentless, or sutureless valves) or leaflet tissue (porcine or bovine pericardial). Tissue leaflets are commonly attached to the internal side of the stent elements, although Trifecta™ (Abbott, Minneapolis, MN, USA) and Mitroflow (LivaNova, London, UK) have externally mounted leaflets. Each valve has a unique appearance on fluoroscopy, which might ease to different degrees of THV positioning in the case of ViV procedure. Of note, no consensus is present for surgical bioprosthesis labelling and sizing, so it is key to remember that, according to the type of prosthesis, the real internal diameter of the stent may differ from the manufacturer’s labelled size [31].

### 6.2. Preprocedural and Procedural Aspects

Gathering details regarding the stent frame (height and type) and leaflets (intra-annular versus supra-annular position) is critical when planning ViV. Data on the stent frame will yield information on the possibility of performing bioprosthetic valve fracturing or remodeling. In ViV procedures, the leaflets of the first bioprosthesis are pinned open, creating a covered cylindrical tube called the “neoskirt”. The neoskirt height is determined by the index bioprosthesis leaflet length and position, and might differ according to the type and implantation height of the ViV THV [31].

In general, SEV can be preferred in patients with small valve diameter (<23 mm) at risk of elevated post-procedural gradients or patients with PPM, while BEV in cases of preservation of coronary access is a priority or in cases of small STJ and ascending aorta dimensions [32]. THV choice can today be guided by a dedicated application developed by Vinayak Bapat (Valve-in-Valve app) [33].

### 6.3. Valve Sizing

Measurements of index bioprosthesis can be easily taken on CT before the ViV procedure, and might be performed at different levels when dealing with index THV3. Of note, while in surgical valves the frame remains circular, the stent frame of the index THV tends to adapt to native anatomies, often losing its circular shape. Some degree of THV oversizing is beneficial in redo-TAVR, especially in cases of the non-structural valve deterioration (NSVD) of THV due to PVL [34]. On the other hand, caution should be placed when oversizing intra-annular SEV in BEV, due to the risk of pin-wheeling, and BEV in a SEV, due to the possible overexpansion of index SEV to such an extent that it might not only render coronary access more cumbersome, but also it could increase the risk of coronary obstruction.

### 6.4. Bioprosthetic Valve Fracture or Remodeling

Bioprosthetic valve fracture should be contemplated in smaller bioprostheses (e.g., manufacturer size ≤ 23 mm and in porcine valves ≤ 25 mm), when feasible (Medtronic Mosaic, Edwards Lifesciences Perimount and Magna, or Sorin Mitroflow). On the other hand, remodeling can be contemplated for some stented (e.g., Inspiris Resilia, Edwards, Irvine, CA, USA) and sutureless bioprostheses and THVs (e.g., CoreValve, Irvine, CA, USA). The timing of bioprosthetic valve fracture can be before TAVR, which implies easier THV implantation at the expense of potential temporary hemodynamic instability due to iatrogenic aortic regurgitation, or after TAVR, at the price of a higher risk of valve migration, embolization, or leaflet injury [32].

### 6.5. Clinical Outcomes after ViV

Overall, patients undergoing ViV procedures experience lower in-hospital mortality, but similar medium-term outcomes when compared with those undergoing redo SAVR/surgical explant [35]. When comparing transcatheter procedures in observational studies, equipoise in terms of clinical outcomes has been suggested between native TAVR, ViV after SAVR, and ViV after TAVR [36,37]. When compared with native TAVR, although ViV procedures have a lower risk of annular injury, PVL (in case of stented surgical prosthesis), permanent pacemaker implantation, ViV in stented surgical valves, and BEV may carry a higher risk of residual elevated gradient, while ViV in stentless valves has a higher rate of coronary obstruction [31].

## 7. Challenges Associated with TAVR

Although the efficacy of the TAVR procedure has been proven in patients with aortic valve stenosis, it is associated with varying types of complications such as stroke, conduction disturbances, paravalvular leak, and coronary overlap that may increase the length of stay and readmission rate associated with the procedure. Advancements in implantation techniques, such as the cusp-overlap view for self-expanding THV platforms and the high deployment technique (HDT) for balloon-expandable THV, have significantly reduced the occurrence of conduction abnormalities [38,39]. Moreover, newer THV designs with improved sealing skirts have progressively decreased the incidence of more-than-mild paravalvular leak, as evidenced by rates below 1% in the PARTNER 3 trial and 3.5% in the Evolut Low Risk trial [40,41]. Despite improvements in device technology and increased operator experience, challenges persist with respect to stroke and coronary overlap in the TAVR procedure.

### 7.1. Stroke

Stroke is one of the devastating complications of the TAVR procedure, with a varying rate of 4–6% in high-risk patients and 0.6% to 3.4% in the latest trials in low-risk patients [42]. The Sentinel cerebral embolic protection (CEP) device (Boston Scientific) is the only FDA-approved and commercially available device. Although data from MISTRAL-C and CLEAN-TAVI suggested a lower incidence of subclinical brain injuries assessed by DW-MRI with use of CEP devices, the PROTECTED TAVR trial data showed that the routine use of a CEP device does not result in a lower risk of clinical stroke within 72 h among patients undergoing transfemoral TAVR for aortic stenosis [43,44,45]. The ongoing BHF PROTECT-TAVI (British Heart Foundation Randomized Trial of Routine Cerebral Embolic Protection in Transcatheter Aortic Valve Implantation; ISRCTN Registry number, ISRCTN16665769) will further address the evidence gap. The primary outcome is all-cause stroke through 72 h post TAVR or discharge in an estimated patient population of 7730 patients with a primary completion date of 2025 [45].

Other multiple CEP devices have been developed to either capture or deflect emboli during the TAVR procedure, as shown in the Table 3 [46]. The ProtEmbo device is unique in providing coverage to all three arch vessels, can be delivered through 6 French left radial access and has the smallest pore size of 60 micrometer. Recently, the PROTEMBO Pivotal IDE trial (NCT 05873816) obtained FDA approval and is set to enroll 250–500 patients. The study aims to compare the ProtEmbo device, with half of the patients receiving no CEP device and the other half receiving the Sentinel device (Boston Scientific) [47]. The PROTECT Head-to-Head study (PROTECTH2H) (NCT05684146) is an ongoing prospective, randomized, open label, multicenter study to evaluate the safety and efficacy of Emboliner EPD compared to the control device (Sentinel CPS) in terms of 30-day composite major adverse cardiac and cerebrovascular events (MACCE events) following the TAVR procedure [47].

### 7.2. Coronary Access

As TAVR expands to younger and lower risk patients, preserving the ability to selectively cannulate the coronary ostia is an important concern. As compared to SAVR, the displacement of the native valve leaflets, commissural misalignment, and supra-annular THV platform where leaflets that extend above the coronary ostia may make coronary access (CA) challenging [48]. Implantation techniques to improve THV commissural alignment with current THVs (specific flush port positioning) and leaflet modification techniques, including bioprosthetic or native aortic scallop intentional laceration to prevent iatrogenic coronary artery obstruction (BASILICA), have shown success in improving the chances of coronary access following TAVR [48]. The ALIGN-ACCESS study evaluated the impact of commissural alignment on the feasibility of CA after TAVR. Final valve orientation was favorable to commissural alignment in 85.9% of Evolut and 69.4% of ACURATE neo cases. Selective CA was higher for Sapien 3 than for aligned and misaligned supraannular THVs (95% versus 71% versus 46%, *p* < 0.001) [49].

### 7.3. Coronary Obstruction Risk Evaluation and Management during Valve-in-Valve Procedures

The main risk factors for this complication are outlined in Table 4, and are mainly related to the interference of the valve complex with the anatomy of the coronary ostium and sinus of Valsalva. The mitigation of coronary obstruction risk entails, first, appropriate THV selection, e.g., a short-frame THV might be preferably implanted within a tall-frame THV in order to reduce the neoskirt height. Second, additional techniques might be considered as alternatives to snorkel/chimney stenting, such as bioprosthetic or native aortic scallop intentional laceration to prevent iatrogenic coronary artery obstruction (BASILICA) [50], balloon-assisted BASILICA [51], and CATHeter Electrosurgical Debulking and RemovAL (CATHEDRAL) [51]. Recently published data has evaluated the role of real-time transesophageal echocardiography, coupled with color Doppler and pulsed-wave Doppler techniques, as a modality to assess immediate coronary artery patency and to identify asymptomatic coronary obstruction during ViV TAVR [52].

## 8. Future Indications

### 8.1. Asymptomatic AS

Current society guidelines only recommend aortic valve replacement for patients with symptomatic severe AS, with SAVR being considered (class IIa, level B) in asymptomatic patients with very severe AS (mean gradient > 60 mmHg, peak velocity > 5 m/s), left ventricular systolic dysfunction, or markedly elevated BNP (3× upper limit of normal) on repeat measures [5]. However, intervention for asymptomatic severe AS not meeting these criteria remains controversial. Two recent studies, namely the Aortic Valve ReplAcement Versus Conservative Treatment in Asymptomatic SeveRe Aortic Stenosis (AVATAR) and the Randomized Comparison of Early Surgery versus Conventional Treatment in Very Severe Aortic Stenosis (RECOVERY), have challenged this traditional approach to managing aortic stenosis [53,54]. The AVATAR trial (*n* = 157) was a randomized parallel trial that compared early SAVR and conservative management for asymptomatic patients with severe AS. Notably, in this trial, asymptomatic status was confirmed by exercise testing, and patients were excluded if they had very severe AS (peak velocity > 5.5 m/s), reduced left ventricular systolic function (<50%), or high surgical risk (STS Score > 8%). At a median follow-up of 32 months, the patients who underwent early SAVR were significantly less likely to experience the primary outcome of all-cause death, heart failure, acute myocardial infarction, or stroke (15.2% vs. 34.7%, *p* = 0.02) [53]. These findings built on the earlier Korean RECOVERY trial (*n* = 145), which found early surgery to be associated with a lower operative or cardiovascular mortality at 4 years compared to watchful waiting (1% vs. 6%, *p* < 0.05). The patients in the later study had true severe aortic stenosis (aortic valve area ≤ 0.75 cm^2^ plus either a velocity ≥ 4.5 m/s or mean gradient ≥ 50 mmHg) and preserved LV systolic function but did not undergo exercise testing [54]. In the transcatheter field, the Evaluation of TAVR Compared to Surveillance for Patients With Asymptomatic Severe Aortic Stenosis (EARLY TAVR) (NCT03042104) trial has just completed enrolment (*n* = 901) and will assess the impact of treating asymptomatic patients with the Edwards SAPIEN 3/S3 Ultra valve.

### 8.2. Moderate AS

An even greater deviation from current practice is intervening with TAVR for moderate symptomatic aortic stenosis rather than a ‘watch and wait’ approach. This concept has evolved as evidenced by a limited number of studies such as VALVENOR, which indicated that patients with symptomatic moderate AS have higher mortality than patients with mild AS [55,56,57]. Notably, there was no difference in mortality between asymptomatic moderate AS and mild AS in this study. A number of trials are currently comparing TAVR plus optimal medical therapy to optimal medical therapy alone in symptomatic moderate AS. The EXPAND TAVR II Pivotal trial [NCT05149755] is currently randomizing patients with symptomatic moderate AS to either treatment with a Medtronic Evolut Pro+/FX valve or medical treatment alone and has an estimated primary completion date of February 2026. Similarly, the PROGRESS randomised control trial [NCT04889872] will evaluate clinical outcomes in the same patient cohort using the Edwards SAPIEN 3/S3 Ultra valve and has an estimated primary completion date of 2029. Finally, the Transcatheter Aortic Valve Replacement to Unload the Left Ventricle in Patients with Advanced Heart Failure (TAVR-UNLOAD) [NCT02661451] will compare TAVR (Edwards Sapien3/S3 Ultra) to optimal heart failure treatment in patients with moderate AS and left ventricular systolic dysfunction (LVEF 20–50%). The earlier treatment of aortic stenosis (asymptomatic severe/moderate AS) has a myriad of potential benefits, including improved functional performance, reduced sudden cardiac death risk, less left ventricular hypertrophy and remodelling, reduced left atrial dilatation and risk of atrial fibrillation. However, these perceived benefits need to be balanced with procedural risk (death, stroke, vascular complications, need for pacing) and potential complications during follow-up (bioprosthetic failure, periprosthetic leak, infective endocarditis) [58,59] Furthermore, at present, there is a paucity of data to support intervention for moderate AS and is not within guideline recommendations.

### 8.3. Aortic Regurgitation

In contrast to SAVR, TAVR is not indicated for the treatment for aortic regurgitation (AR). Patients with isolated AR were excluded from the pivotal TAVR clinical trials, hence TAVR never carried an indication for AR. In addition, anatomical features intrinsic to AR (lack of annular calcification, large annuli, concomitant aortopathy) impair valve anchoring and increase the risk of valve embolization. Furthermore, the traditional practice of oversizing current transcatheter heart valves to treat AR has been associated with increased rates of aortic root injury and the need for pacemaker implantation [60]. Despite these considerations, TAVR for native pure AR accounted for almost 40% of off-label TAVR cases entered in the STS/ACC TVT Registry between 2011 and 2014 [61]. This is likely driven by an unmet clinical need, with almost 8% of patients with AR and a clinical indication for SAVR foregoing the procedure due to advanced age, frailty, and overall operative risk [61]. Jena Valve has developed an AR-specific transcatheter heart valve (Trilogy Valve) which has CE Mark approval. This short-frame, self-expanding device has unique clipping apparatus called “locators” that attach behind the valve leaflets, which fixates the valve at the leaflet level and ensures commissural alignment. Early data from the ALIGN-AR study (symptomatic ≥3+ AR and high surgical risk) has shown successful implantation in 95.7% of the patients and encouraging clinical outcomes at 30 days, with the exception of a high pacemaker rate (21.1%) [62]. A separate valve for AR has been developed by JC Medical (J-Valve). This device uses U-shaped “anchor rings” that sit within the coronary sinuses to stabilize the valve and facilitate accurate positioning. In a Chinese population with either AS (*n* = 63) or AR (*n* = 44), the device was successfully implanted in 91.5% of the patients, and 97.6% of the patients had mild or less than mild paravalvular leak at a 2-year follow-up [63]. An early feasibility study using the J-Valve Transfemoral System for North American patients commenced enrolment in October 2023.

## 9. Conclusions (VD)

Twenty years after its first procedure, TAVR has pushed the boundaries of the management of AS across the entire spectrum of surgical risk patients. With the extension of indications to patients at lower risk, further efforts should aim to refine appropriate valve selection, establish the durability of TAVR devices, and to plan treatment strategies for the life time management of AS in younger low-risk patients.

## Figures and Tables

**Table 1:** Current indications of TAVR.

2020 ACC/AHA Guidelines [4]	2021 ESC Guidelines [5]
Transfemoral TAVR is recommended over SAVR in patients aged >80 years or in younger patients with a life expectancy <10 years and no anatomic contraindication to transfemoral TAVR. (Class IA)	TAVR is recommended in older patients with age >75 years or those at high risk (STS PROM/EuroSCORE II > 8%) or unsuitable for surgery. (Class IA)
TAVR is recommended for patients with symptomatic severe AS aged 65–80 years and no anatomic contraindication to transfemoral TAVR. (Class IA)	SAVR or TAVR is recommended for remaining patients after taking into account of patient’s clinical, anatomic, and procedural characteristics. (Class IB)

**Table 2:** Currently available TAVR prosthesis.

Expansion Mechanism	Prosthesis	Valve Height (mm)	Leaflet Position	Frame Cell Size	Outer Seal	Access	Sheath			Delivery System			Repositionable	Retrievable
							ID/OD (French)	Integrated	Expandable	OD (French)	Flexibility	Steerability		
SEV	CoreValve	53–55	Supra-annular	+	-	TV, TAo	Variable	-	Variable	18	-	-	+	+
	Evolut R	45–46	Supra-annular	+	-	TV/TAo	14/18, 16/20 (34 mm)	+	-	14, 16	-	-	+	+
	Evolut PRO	45	Supra-annular	+	+	TV/TAo	16/20	+	-	16	-	-	+	+
	Evolut PRO+	45–46	Supra-annular	+	++	TV	14/18, 16/20 (34 mm)	+	-	14, 16	-	-	+	+
	Acurate TA	44–46	Intra-annular	+++	-	TA	-	-	-	28	-	-	-	-
	Acurate Neo	48–51	Supra-annular	+++	+	TV, TA	14/23	-	+	18	+	-	-	-
	Acurate Neo 2	48–51	Supra-annular	+++	++	TV, TA	14/23	-	+	14	+	-	-	-
	Portico	47–51	Intra-annular	++	+	TV, TAo	14/18, 15/19 (27, 29 mm)	+	-	18, 19	++	-	+	+
	Navitor	47–48	Intra-annular	++	++	TV	14/18, 15/19 (27, 29, 35 mm)	+	-	14, 15	++	-	+	+
	Hydra	51–55	Supra-annular	+++	+	TV	18/NA	-	-	18	+	-	+	+
	Allegra	37–43	Supra-annular	+	-	TV	18/20.4	-	-	18	+	-	+	+
	JenaValve	NA	Supra-annular	+++	-	TA	-	-	-	32	-	-	+	-
	Trilogy	NA	Supra-annular	++	+	TV	18	-	-	18	+	+	+	-
	J-Valve	NA	Intra-annular	NA	-	TV/TA	NA	-	-	18	-	+	-	-
	Venus-A	NA	Supra-annular	+	+	TV	NA	NA	NA	19	NA	NA	+	-
	VitaFlow	NA	Supra-annular	++	++	TV	NA	NA	NA	16/18	NA	NA	+	-
	TaurusOne	NA	Supra-annular	++	+	TV	NA	NA	NA	18	NA	NA	+	-
BEV	Sapien	14–16	Intra-annular	+	-	TV/TA/TAo	22/26, 24/28 (26 mm)	-	+	22, 24	-	-	-	-
	Sapien XT	14–19	Intra-annular	+	-	TV/TA/TAo	16/20, 18/22 (26 mm), 20/24 (29 mm)	-	+	16, 18, 20	-	-	-	-
	Sapien 3	15–22	Intra-annular	++	+	TV/TA/TAo	14/17.4, 16/20 (29 mm)	-	+	18, 21	-	+	-	-
	Sapien 3 Ultra	15–20	Intra-annular	++	++	TV	14/17.4	-	+	18	-	+	-	-
	Sapien 3 Ultra Resilia	15–20	Intra-annular	++	++	TV	TV	-	-	-	-	-	-	-
	Sapien X4	15–20	Intra-annular	++	++	TV	TV	-	+	18	-	+	-	-
	MyVal	17–21	Intra-annular	++	++	TV	14/17.4	-	+	14	-	+	-	+
	MyVal	17–21	Intra-annular	++	++	TV	14/17.4	-	+	14	-	+	-	+
	MyVal Octacor	17–21	Intra-annular	++	++	TV	14/17.4	-	+	14	-	+	-	+
MEV	Lotus	19	Intra-annular	+	++	TV, TAo	18/22, 20/24 (25, 27 mm)	-	-	18, 20	-	-	++	++
	Lotus Edge	19	Intra-annular	+	++	TV, TAo	15/23.7	-	+	22	+	-	++	++

BEV = balloon-expandable valve; ID = internal diameter; MEV = mechanically-expandable valve; OD = outer diameter; SEV = self-expandable valve; TA = transapical; TAo = transaortic; TV = transvascular. Frame cell size and outer seal height is described in ascending order as -, +, ++, +++. Presence or absence of sheath expandability, repositionability, and retrievability are indicated as follows: (+) for presence and (-) for absence.

**Table 3:** Summary of various cerebral embolic protection (CEP) devices. * Ipsilateral access, same site as the transcatheter device delivery system site.

	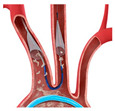	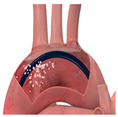	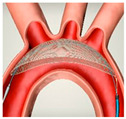	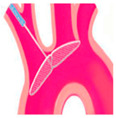	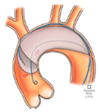	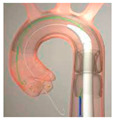	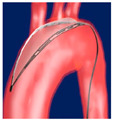	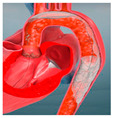	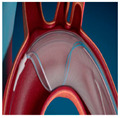
	SENTINEL (Boston Scientific, Marlborough, MA, USA)	ProtEmbo (Protembis, Aachen, Germany)	TriGUARD 3 (Keystone Heart, Tampa, FL, USA)	Embrella (Edwards Lifesciences, Irvine, CA, USA)	Emblok (Innovative Cardiovascular Solutions, Grand Rapids, MI, USA)	Emboliner (Emboline, Santa Cruz, CA, USA)	Point-Guard (Transverse Medical Inc., Denver, CO, USA)	CAPTIS (Filterlex Medical, Caesarea, Israel)	FLOWer (Aortic Lab, Turin, Italy)
Vessels covered	2 vessels	3 vessels	3 vessels	2 vessels	3 vessels	3 vessels	3 vessels	3 vessels	3 vessels
Access site	Radial	Left Radial	Femoral	Radial	Femoral	Femoral	Femoral	Femoral	Femoral
Sheath size	6 French	6 French	8 French	6 French	11 French	10 French	10 French	16 French *	12 French
Pore size (µm)	140	60	145	100	100	150	105	115 × 145	70
Mechanism	Capture	Deflector	Deflector	Deflector	Capture and removal	Capture and removal	Deflector	Capture and removal	Capture and removal
FDA approval/CE marked	FDA/CE	CE	CE	CE	SIH	CE	SIH	FIH	FIH

CE: European conformity; FDA: U.S Food and Drug Administration; FIH: first-in-human; SIH: second-in-human.

**Table 4:** Factors affecting the risk of coronary obstruction during TAVR procedure.

Anatomy	
STJ diameter	Narrow
SoV height	Short
SoV width	Narrow
Coronary height	Low
VTC	<4 mm
VTSTJ	<2.5 mm
Device/procedure	
THV type	Supra-annular
THV implantation depth	High
THV commissural alignment	Misalignment
Previous THV	
THV type	Tall stent frame, supra-annular
Index THV commissural alignment	Misalignment
Previous surgical valve	
Surgical valve type	Supra-annular
Surgical valve leaflet length	Long leaflets

STJ = sinotubular junction; SoV = sinus of Valsalva; THV = transcatheter heart valve; VTC = virtual transcatheter heart valve to coronary distance; VTSTJ = virtual transcatheter heart valve to sinotubular junction distance.
